# The association between epicardial adipose tissue and non-alcoholic fatty liver disease: A systematic review of existing human studies

**DOI:** 10.17179/excli2021-3815

**Published:** 2021-06-14

**Authors:** Hadi Emamat, Hadith Tangestani, Mojgan Behrad Nasab, Hamid Ghalandari, Azita Hekmatdoost

**Affiliations:** 1 Student Research Committee, Department of Clinical Nutrition and Dietetics, Faculty of Nutrition Sciences and Food Technology, National Nutrition and Food Technology Research Institute, Shahid Beheshti University of Medical Sciences, Tehran, Iran; 2 Department of Nutrition, Faculty of Health and Nutrition, Bushehr University of Medical Sciences, Bushehr, Iran; 3 Nutritionist, Emam Reza Hospital, AJA University of Medical Sciences, Tehran, Iran; 4 Department of Clinical Nutrition, Faculty of Nutrition and Food Sciences, Shiraz University of Medical Sciences; 5 Department of Clinical Nutrition and Dietetics, Faculty of Nutrition Sciences and Food Technology, National Nutrition and Food Technology Research Institute, Shahid Beheshti University of Medical Sciences, Tehran, Iran

**Keywords:** epicardial adipose tissue, epicardial fat, NAFLD, non-alcoholic fatty liver disease, visceral fat

## Abstract

The prevalence of non-alcoholic fatty liver disease (NAFLD) has significantly risen all around the world. Although visceral fat mass has been identified as an independent risk factor for NAFLD, the association of other ectopic fat depots, such as Epicardial adipose tissue (EAT), with the disease has not been fully elucidated. The aim of the current study was to systematically review all available human studies conducted on the associations between EAT and NAFLD. All human studies published in English, which examined the association between the thickness or the volume of EAT and the incidence of NAFLD were systematically searched on PubMed, Scopus, and Google Scholar search engines, from inception up to April 2021. Eighteen studies that met inclusion criteria were included in the final review. A total of 86 studies were found through searching the databases. After excluding duplicates, seventy six remained studies were scanned by title and abstract, out of which, 58 were excluded. Finally, eighteen articles (thirteen cross-sectional studies and five case-control studies) published between 2008 and 2021, were included in the review. According to the results of the reviewed articles, EAT was associated with the presence and progression of NAFLD. Furthermore, NAFLD patients with thicker EAT may need a more intensive hepatic follow-up. However, we suggest further investigation to find out the underlying mechanisms describing the observed association.

## Introduction

Epicardial adipose tissue (EAT) is a distinct type of ectopic visceral adipose tissue existing in considerable amounts around the sub-epicardial coronary arteries (Mazurek et al., 2003[26]; Nerlekar et al., 2020[29]). The EAT is located in a layer between the pericardium and the myocardium tissues surrounding the heart (Hirata et al., 2011[9]). It plays various metabolic, thermogenic, and mechanical (cardio-protective) roles in physiological terms (Iacobellis, 2015[10]). Increased cardiac visceral fat, particularly the EAT, has been proposed as a new cardio-metabolic risk factor (Iacobellis et al., 2005[13]), so that measuring its thickness or volume has been suggested as a reliable indicator of visceral adiposity and a potential tool for diagnosing people with metabolic syndrome (MS) (Iacobellis et al., 2003[11]; Iacobellis and Leonetti, 2005[14]).

The prevalence of non-alcoholic fatty liver disease (NAFLD) has grown worldwide, affecting up to 30 % of the general population (Dowman et al., 2010[6]). In recent years, the global growth of obesity and the consequent increase in diabetes have been associated with an increase in the prevalence of NAFLD (Younossi et al., 2018[44]). Although visceral fat mass has been identified as an independent risk factor for NAFLD (van der Poorten et al., 2008[41]; Perseghin, 2011[31]), the association of other ectopic fat depots, such as EAT, with this disease has not been fully elucidated. So far, several observational studies have been conducted in this field indicating a positive association between EAT and NAFLD (Fracanzani et al., 2016[7]; Kim et al., 2016[18]; Colak et al., 2012[5]); however, the results have not always been consistent (Psychari et al., 2016[33]).

The aim of the current study was to systematically review all available human studies on the associations between the EAT and NAFLD.

## Material and Methods

### Data source and search strategy 

This systematic review was conducted according to the Preferred Reporting Items for Systematic Reviews and Meta-analyses (PRISMA) guidelines (Moher et al., 2009[28]). Comprehensive search strategies were used to identify reports of human studies indexed in PubMed, Scopus, and Google Scholar search engines (from inception up to April 2021). The keywords were used to search studies relevant to our study objectives were: ("epicardial fat"[tiab^Note 1^] OR "epicardial adipose"[tiab]) AND ("fatty liver"[Mesh] OR "fatty liver"[tiab] OR NAFLD [tiab] OR NASH [tiab] OR steatosis[tiab] OR steatohepatitis[tiab] OR "hepatic triglyceride"[tiab]). Moreover, the reference lists of the obtained studies were manually verified to find more related studies.

### Study selection

All human studies published in English investigating the association between EAT thickness or volume and the incidence of NAFLD were included. The condition in which the results of a study were reported in more than one article, the one reporting the most complete results was included in the review.

The following study patterns were excluded: 1) not original research (reviews, editorials, non-research letters); 2) case reports or case series; 3) ecologic studies; 4) cell-culture or experimental (animal) studies.

Two reviewers (H. E. and H. T.) independently screened the title and the abstracts of obtained studies to detect potentially eligible ones. A third reviewer (A. H.) made the final decision about any discrepancies raised between reviewers.

### Data extraction and quality assessment 

A data extraction of the following information was conducted: the first author's name, publication year, study origin, study design, sample size, participants' age and gender, imaging system, EAT thickness or volume, and findings. The Newcastle-Ottawa scale was used to assess the quality of included studies. Selection of the study group (maximum 4 stars), quality of the adjustment for confounders (maximum 2 stars), and assessment of outcome (maximum 3 stars) were evaluated (Wells et al., 2008[42]).

## Results

### Search results and study selection 

**Figure 1:**
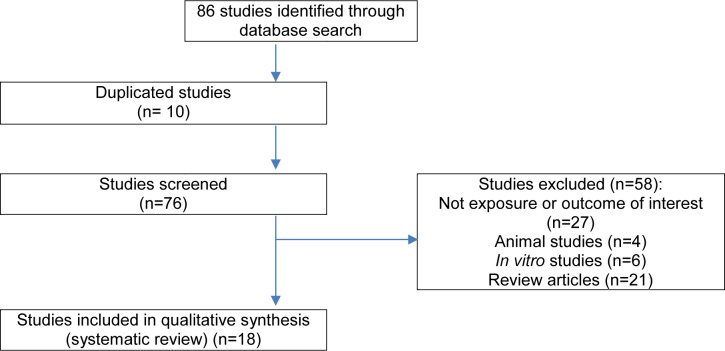
Flowchart of identification of included studies

The flowchart diagram of the selection process is depicted in Figure 1(Fig. 1). Out of 86 articles, 10 were duplicates and were excluded. Overall, 76 studies were screened. A total of 58 articles were excluded because they did not meet the inclusion criteria. Finally, 18 articles (thirteen cross-sectional studies (Fracanzani et al., 2016[7]; Brouha et al., 2018[3]; Cho et al., 2017[4]; Kim et al., 2016[18]; Psychari et al., 2016[33]; Petta et al., 2015[32]; Granér et al., 2015[8]; Topuz et al., 2014[39]; Turan, 2020[40]; Lai et al., 2012[19]; Ledda et al., 2021[21]; Iacobellis et al., 2008[15]; Meng et al., 2018[27]) and five case-control studies (Iacobellis et al., 2014[12], Colak et al., 2012[5], Yilmaz et al., 2011[43], Sunbul et al., 2014[37], Oğuz et al., 2016[30]), published between 2008 and 2021, which met our inclusion criteria were reviewed. Out of these, fourteen studies measured EAT thickness using echocardio-graphy; in the rest, EAT volume was assessed using CT or MRI methods (Brouha et al., 2018[3]; Granér et al., 2015[8]; Ledda et al., 2021[21]; Meng et al., 2018[27]). The characteristics of studies are shown in the Table 1(Tab. 1) (References in Table 1: Brouha et al., 2018[3]; Cho et al., 2017[4]; Colak et al., 2012[5]; Fracanzani et al., 2016[7]; Granér et al., 2014[8]; Iacobellis et al., 2008[15]; Iacobellis et al., 2014[12]; Kim et al., 2016[18]; Lai et al., 2012[19]; Ledda et al., 2021[21]; Meng et al., 2018[27]; Oğuz et al., 2016[30]; Petta et al., 2015[32]; Psychari et al., 2016[33]; Sunbul et al., 2014[37]; Topuz et al., 2014[39]; Turan, 2020[40]; Yilmaz et al., 2011[43]).

### Main results

#### Case-control studies

Five of the studies were case-control studies. Iacobellis et al. (2014[12]) showed that EAT thickness in obese people with NAFLD was significantly higher than healthy obese people (p < 0.01). Moreover, the EAT thickness in patients with severe hepatic steatosis was higher than those with moderate steatosis (9.7 ± 0.2 and 8 ± 0.7 mm, respectively; p < 0.01). Colak et al. (2012[5]) observed that the EAT thickness was significantly higher in patients with NAFLD compared to the age- and sex-matched controls (0.58 ± 0.18 and 0.36 ± 0.17 cm, respectively; p < 0.001). Similarly, Yilmaz et al. (2011[43]) reported that the thickness of EAT in the control group was significantly lower than NAFLD group (0.54 ± 0.10 and 0.64 ± 0.13 cm, respectively; p < 0.001). EAT thickness was significantly higher in NAFLD patients than in controls in the research conducted by Sunbul et al. (0.32 ± 0.06 and 0.26 ± 0.04 mm, respectively; p < 0.001) (Sunbul et al., 2014[37]). Finally, Oğuz et al. (2016[30]) reported that NAFLD patients have a significantly higher EAT thickness (0.51 ± 0.25 vs. 0.29 ± 0.09 cm, p < 0.001) than controls. Therefore, these studies consistently indicated that the thickness of EAT is markedly higher in patients with NAFLD, compared to healthy and/or non-NAFLD individuals.

#### Cross-sectional studies 

Thirteen cross-sectional studies were reviewed. Fracanzani et al. (2016[7]) reported that the mean value of EAT in subjects with or without non-alcoholic steatohepatitis (NASH) (5.9 ± 2.5 and 4.0 ± 2.4, respectively; p = 0.001) and with or without fibrosis score > 2 (6.9 ± 2.3 and 4.7 ± 2.5, respectively; p = 0.0001) was significantly different. In another study in diabetic individuals without known CHD, the EAT volume was significantly higher in NAFLD patients compared to non-NAFLD patients (126.5 ml and 85.4 ml, respectively; p = 0.002). Additionally, both liver fat content and liver fibrosis positively correlated with EAT volume (Brouha et al., 2018[3]). Cho et al. (2017[4]) found that NAFLD patients, especially those with MS, have higher EAT-t (thickness) in comparison to subjects without NAFLD (MS with NAFLD, EAT-t = 7.5 ± 4.4 mm; MS without NAFLD, EAT-t =4.9 ± 3.0 mm; non-MS with NAFLD, EAT-t =5.9 ± 3.6 mm; and non-MS without NAFLD, EAT-t =4.4 ± 3.5 mm, p < 0.001). Kim et al. (2016[18]) reported that the EAT thickness in NAFLD group was significantly higher than in non-NAFLD individuals (3.27 mm vs 3.07 mm, p = 0.003). Moreover, EAT was significantly associated with the incidence of NAFLD (OR= 1.705 [CI: 1.211, 2.40]). In addition, EAT was higher in subjects with severe *vs.* milder fibrosis (8.5 ± 3.0 vs. 7.2 ± 2.3 mm; p = 0.006) (Petta et al., 2015[32]). Granér et al. observed that the EAT volume increased with increasing amount of liver fat (p < 0.001) in non-diabetic men (Granér et al., 2015[8]). Among participants who were hospitalized for coronary angiography, those with NAFLD have thicker EAT than non-NAFLD patients (0.90 ± 0.19 cm and 0.58 ± 0.18 cm, respectively; p < 0.001) (Topuz et al., 2014[39]). Another study stated that NAFLD Fibrosis Score (NFS) was positively correlated with EAT (r= 0.224, p = .019) (Turan, 2020[40]). In asymptomatic participants enrolled in a cardiovascular health survey, there was an orderly increase in EAT thickness as fatty liver severity increased (p < .001) (Lai et al., 2012[19]). Eufrasia Ledda et al. showed that the EAT volume positively correlated with mean liver density (MLD) (p = 0.368, p < 0.001). Furthermore, the EAT volume was significantly higher in the group with hepatic steatosis (HS) (p < 0.001) (Ledda et al., 2021[21]). In another study, EAT was correlated with AST (aspartate amino-transferase)/ALT (alanine amino-transferase) ratio (r = 0.77, p < 0.01), ALT (r = 0.58, p < 0.01), and AST (r = 0.56, p < 0.01) in HIV^+^MS^+^ subjects (Iacobellis et al., 2008[15]). Another study was able to show that NAFLD patients have higher EAT volume than healthy individuals (Meng et al., 2018[27]). Psychari et al. stated that the EAT was not thicker in NAFLD patients; however, it was positively related to indices of insulin resistance and inflammation (Psychari et al., 2016[33]).

## Discussion

The aim of the present study was to summarize the existing evidence on the association between EAT and NAFLD conducted on human subjects. The pooled data of the existing literature postulate that the increased EAT might be associated with the presence and progression of NAFLD and/or its related indicators such as insulin resistance or inflammation. 

The majority of included studies consistently showed a positive association between EAT and NAFLD. Only one study failed to show a positive association between epicardial fat thickness and NAFLD (Psychari et al., 2016[33]). However, researchers in the latter study were able to detect a positive correlation between epicardial fat thickness and the indices of insulin resistance and inflammation, both of which could be counted as underlying triggers in the pathogenesis of NAFLD (Manco, 2017[24]; Asrih and Jornayvaz, 2013[1]). Increasing evidence shows that visceral adipose tissue is a causative risk factor for fatty liver, rather than overall obesity (Schäffler et al., 2005[35]; Thomas et al., 2005[38]). Epicardial fat is significantly correlated with intra-abdominal visceral fat and can be considered as a measurable indicator instead (Iacobellis et al., 2003[11]). Epicardial fat, similar to other visceral adiposity, acts as an endocrine or paracrine organ and produces proinflammatory adipokines and interleukins such as vaspin, TNF-α, interleukin-6, interleukin-17, and angiotensin (Lana et al., 2016[20]); all of which are involved in the development and progression of cardiovascular and fatty liver disease (Şengül and Özveren, 2013[36]). On the other hand, the epicardial fat releases almost twice as much fatty acids as other fat depots such as the perirenal and pericardial depots (Marchington et al., 1989[25]). It has been observed that epicardial fat correlates with free fatty acid levels in humans (Kankaanpää et al., 2006[17]), leading to increased flux of free fatty acids to the liver, which disrupts the function of hepatocytes in the management of fats, inducing lipotoxicity and NAFLD (Li et al., 2018[22]). Additionally, the existing literature shows the EAT is associated with hepatic steatosis (Iacobellis et al., 2014[12], Brouha et al., 2018[3], Granér et al., 2015[8], Fracanzani et al., 2016[7]), fatty liver index (Fracanzani et al., 2016[7]), liver fibrosis (Petta et al., 2015[32], Turan, 2020[40]), mean liver density (MLD) (Ledda et al., 2021[21]), and serum liver enzymes (Iacobellis et al., 2008[15]). Iacobellis et al. were even able to show that the correlation between EAT and liver steatosis is stronger than that of BMI or waist circumference (R^2^ = 0.77, p < 0.001) (Iacobellis et al., 2014[12]). Therefore, we can speculate that the increase in EAT might increase the incidence of NAFLD. According to a systematic review, EAT thickness > 5 mm, or a volume > 125 mL or 68 mL/m^2^ might be considered as a risk factor for metabolic syndrome and coronary artery disease (Bertaso et al., 2013[2]). However, further studies are needed to determine exactly how much an increase in epicardial fat worsens the progression of NAFLD.

Recently, a meta-analysis was conducted to examine the same possible correlation. This study, which included 13 observational studies, showed that EAT was higher in subjects with NAFLD than in non-NAFLD subjects (EAT, SMD: 0.73, 95 % CI 0.51-0.94, p < 0.001) (Liu et al., 2019[23]). In the current study, we included all human studies and five new studies were taken into account.

In the studies reviewed in this article, EAT thickness and volume measurement were performed using ultrasound echocardiography and Computed tomography (CT) or magnetic resonance imaging (MRI), respectively. CT and MRI, especially, are the gold standard techniques, and accurate methods to estimate visceral fat and have high spatial resolution, which make them suitable for volumetric assessments (Bertaso et al., 2013[2]). Nevertheless, they are expensive and CT requires radiation exposure. Recently, the ultrasound technique has come into play as a cheap and easy-to-perform method (Iacobellis et al., 2003[11]). This method is valid, safe, easily reproducible, non-invasive, and can be routinely implemented (Salazar et al., 2016[34]; Iacobellis et al., 2003[11]). Echocardiographic EAT clearly shows visceral fat mass rather than general obesity. It correlates with metabolic syndrome, insulin resistance, coronary artery disease, and atherosclerosis; therefore, it might serve as a valuable tool for cardiometabolic risk assessment (Iacobellis and Willens, 2009[16]).

## Conclusion

In summary, evidence shows that EAT is associated with the presence and progression of NAFLD. EAT is also associated with serum liver enzymes concentration, hepatic steatosis, and fibrosis in NAFLD patients. Furthermore, NAFLD patients with higher EAT may need a more intensive hepatic follow-up. Then, EAT measurement can be used as a prognostic indicator for NAFLD. However, further studies are needed to determine exactly how much an increase in EAT worsens the progression of NAFLD.

## Notes

^1^ truncation character (wildcard) [**tiab**] = limit to title or abstract

## Acknowledgements

This study is related to the project NO. 1398/10133 from Student Research Committee, Shahid Beheshti University of Medical Sciences, Tehran, Iran. We also appreciate the “Student Research Committee” and “Research & Technology Chancellor” in Shahid Beheshti University of Medical Sciences for their financial support of this study.

## Disclosure statement

The authors report no conflict of interest.

## Authors' contribution

H.E. and A.H. conceptualized the study and wrote the manuscript. H.T., M.B. and H. G. contributed to drafting of the manuscript. All authors approved the final version of the manuscript.

## Figures and Tables

**Table 1:**
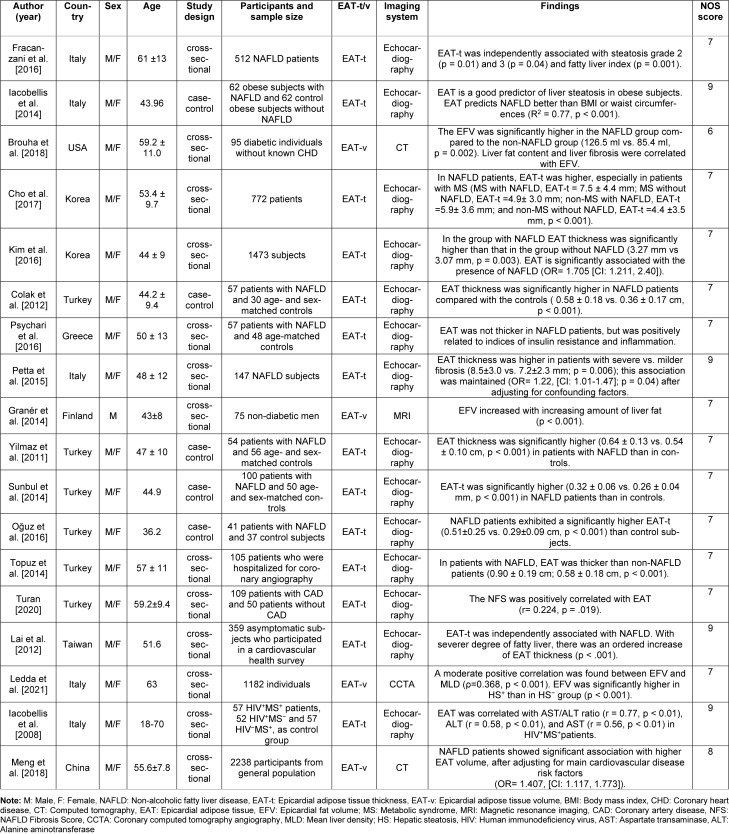
Characteristics of the studies in this systematic review
